# *Burkholderia pseudomallei* Misidentified by Automated System

**DOI:** 10.3201/eid1511.081719

**Published:** 2009-11

**Authors:** Christoph Weissert, Günter Dollenmaier, Philippe Rafeiner, Julia Riehm, Detlev Schultze

**Affiliations:** Institute for Clinical Microbiology and Immunology, St. Gallen, Switzerland (C. Weissert, G. Dollenmaier, D. Schultze); Cantonal Hospital, St. Gallen, (P. Rafeiner); Federal Armed Forces Institute of Microbiology, Munich, Germany (J. Riehm)

**Keywords:** Burkholderia infections, melioidosis, Burkholderia pseudomallei, communicable diseases, emerging infectious diseases, bacteria, dispatch

## Abstract

After returning from Thailand, a 35-year-old man from Switzerland was hospitalized with an abscess of the head. Material cultured from the abscess and adjacent bone grew a gram-negative rod, which was misidentified by an automated microbiology system as *Burkholderia cepacia*. The organism was eventually identified by molecular methods as *B*. *pseudomallei*.

*Burkholderia pseudomallei*, the etiologic agent of melioidosis, can cause pyogenic or granulomatous lesions and is endemic to tropic regions, mainly in Southeast Asia and northern Australia. This organism is a potential category B bioterrorism agent (*1*). Melioidosis occurs sporadically in travelers returning from disease-endemic areas, and laboratories in regions where this disease is not endemic are not familiar with identification of *B*. *pseudomallei*, thus potentially leading to misidentification (*2*). We report the misidentification of this organism by an automated microbiology system.

## The Study

On August 20, 2008, a 35-year-old man from Switzerland was admitted to the Cantonal Hospital in St. Gallen, Switzerland. He had extradural cranial abscess of the right parietal area and a defect in adjacent bone. In May and June 2008, he traveled to Singapore and Malaysia (Kuala Lumpur and the Perhentian Islands), then to southwestern (Ko Samui, Ko Tau) and northern (Chiang Mai) Thailand where he went trekking and river rafting.

The patient did not remember receiving a head injury during his trip. Seventeen days after returning to Switzerland, he had a swelling in the right parietal area of the head. The parietal bulge increased, but puncture by his general practitioner showed no aspirate. During the next 7 weeks, the bulge became painful and secretion of pus was noted at the time of admission.

At admission, his general condition was good and he had no signs of systemic inflammation. He did not have any neurologic deficits or other abnormal findings. Test results for complete blood cell count, C-reactive protein and creatinine levels, and liver functions were normal. Computed tomography and magnetic resonance imaging of the head showed the abscess and a small defect of bone (Figure). The abscess and part of the cranial bone were then surgically removed.

**Figure:**
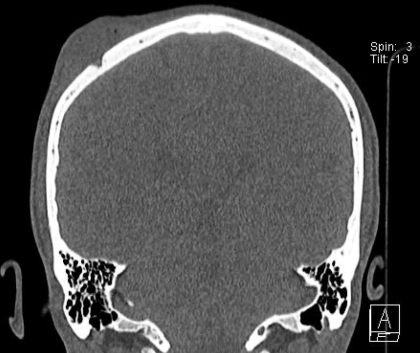
Computed cranial tomography image of the patient showing a swelling at the right parietal area and a small defect of the bone.

Culture material from the abscess and biopsy specimens of the abscess capsule and the cranial bone grew gram-negative, oxidase-positive rods, and smooth creamy colonies on sheep blood agar after incubation for 48 hours at 35°C. For identification, a suspension of the isolate was prepared and tested in a UNMIC/ID-62 panel of the BD Phoenix Automated Microbiology System (Becton Dickinson AG, Allschwil, Switzerland) per the manufacturer’s instructions. This system identified the isolate as *B*. *cepacia* (99% confidence). When a species is identified with >90% confidence, the Phoenix System gives an identification result as a measure of likelihood that the identification is the only correct one.

The patient was discharged from hospital after 5 days with a preliminary diagnosis of *B*. *cepacia* infection of soft tissue (the cranial bone lesion was attributed to trauma). He was treated with oral cotrimoxazole (160/800 mg 2× a day) for 16 days. The isolate was sensitive to cotrimoxazole (MIC = 1 mg/L for trimethoprim and 19 mg/L for sulfamethoxazole) by the Phoenix System for *B*. *cepacia* (Becton Dickinson). The isolate was sensitive to imipenem, ceftazidime, doxycycline, cotrimoxazole, and tetracycline by Etest (AB Biodisk, Solna, Sweden).

The diagnosis was regarded as preliminary for the following reasons. First, identification of *B*. *cepacia* by common automated identification instruments such as the Phoenix System or VITEK 2 (bioMérieux, Geneva, Switzerland) requires confirmatory identification by molecular tests (*3*). Second, an abscess is an uncommon location for *B*. *cepacia* (*4*). Third, the bacterial colonies emitted an unexpected, earthy odor. Fourth, the isolate was sensitive to amoxicillin (MIC = 8 mg/L) and clavulanate (MIC = 4 mg/L).

To verify identification of the isolate, a 500-bp fragment of the 16S rRNA gene was amplified and sequenced by using the Fast MicroSeq 500 16S rDNA Bacterial Identification Kit and a PRISM 310 Genetic Analyzer (both from Applied Biosystems, Foster City, CA, USA) according to the manufacturer’s instructions. Sequence analysis was performed by using MicroSeq ID Microbial Identification Software (Applied Biosystems). The MicroSeq ID 2.0 500-bp library identified *B*. *pseudomallei* (ATCC 23343, gb DQ108392.1) with a 446-bp consensus length and 1 mismatch with *B*. *mallei* (NCTC 10247, gb CP000548.1). These results suggested that our isolate was *B*. *pseudomallei*.

Additional investigations with a flagellin C gene–specific real-time PCR (*5*) suggested that the isolate was *B*. *pseudomallei* or *B*. *mallei*. Multilocus sequence typing (*6*) showed the allelic profile 1/2/3/1/1/2/1, which identified the isolate as *B*. *pseudomallei* sequence type 306. This sequence type was isolated from serum samples of 2 patients in Thailand with invasive melioidosis in 1991 and 2006.

Forty-four days after surgery, new biopsy specimens of soft tissue below the parietal scar and a sample of galea aponeurotica were cultured for *B*. *pseudomallei* after the patient received 2 weeks of inadequate therapy with low-dose cotrimoxazole to determine its drug resistance pattern. Cultures yielded only *Staphylococcus epidermidis*, which was regarded as a contaminant. To ensure eradication of *B*. *pseudomallei*, the patient was treated with imipenem (500 mg, 4×/day), cotrimoxazole (400 mg trimethoprim/day), and leucovorine (15 mg, 3× a week) for 2 weeks and later with cotrimoxazole (320 mg trimethoprim/day) and leucovorine (15 mg, 3×/week) for 6 months. The patient recovered after 6 months; he had a small indentation without signs of inflammation at the site of the abscess.

For comparison with the Phoenix System, we retrospectively analyzed the isolate by using the API 20NE biochemical test panel V7.0 (bioMérieux). This panel identified the isolate as *B*. *pseudomallei* (profile 1156577; 99.9% ID, 1.0 T).

## Conclusions

Automated methods for identification of bacterial isolates and testing of antimicrobial drug susceptibility, such as the Phoenix System, have become standard in most clinical laboratories because they are easy to use and turnaround time is rapid. The Phoenix System uses fluorogenic and chromogenic substrates for its identification algorithms, a broth-based antimicrobial drug–susceptibility testing method, and a data-processing application (Phoenix EpiCenter; Becton Dickinson AG). Unfortunately, *B*. *pseudomallei* was not in the database of this system, which led to misidentification of our isolate as *B*. *cepacia*. Failure to correctly identify *B*. *pseudomallei* has also been reported with another widely used automated system, the Vitek2 system (*7*). To identify isolates from patients traveling to disease-endemic areas, automated systems should be updated for identification of *B*. *pseudomallei* and other rare bacteria that cause severe human infections, are hazardous to laboratory personnel, and may be used as bioterrorism agents (e.g., *Brucella* spp. and *B*. *mallei*).

Three modes of acquisition (inhalation, ingestion, and inoculation) are recognized for *B*. *pseudomallei* infection. Skin and soft tissue infections may occur after minor wounds or from hematogenous spread (*8*). In our patient, inoculation of the skin with *B*. *pseudomallei* after a minor injury in Thailand is probably the mode of infection.

Although laboratory personnel handled cultures of *B*. *pseudomallei* for identification and drug resistance testing and smelled culture plates without knowing the isolate’s identity, none became ill or showed signs of melioidosis. According to expert consensus (*9*), exposure of our laboratory personnel was classified as a low risk. Serum samples were stored at –30°C to enable serologic testing for any subsequent illness.

Laboratories in regions where *B*. *pseudomallei* is not endemic should be aware of misdiagnosis of isolates by automated methods for bacterial identification and antimicrobial drug susceptibility testing. Identification of *Burkholderia* spp. by the Phoenix EpiCenter should be confirmed by molecular methods and by the API 20NE system in suspected cases of *B*. *pseudomallei* infection. Because of its high rate of accuracy and ease of use, the API 20NE system should be used first for any suspected colony when automated systems do not contain the adequate profile (*10*).
